# Graphene oxide with 1-nm-thick adlayer for efficient and near-instant removal of per- and polyfluoroalkyl substances

**DOI:** 10.1093/nsr/nwaf092

**Published:** 2025-03-07

**Authors:** Dingxin Xu, Wenhui Ding, Xinyu Gong, Xianjun Tan, Hang Li, Fei Li, Mingrui Zhang, Yuxiong Huang, Yang Su, Hui-Ming Cheng

**Affiliations:** Institute of Materials Research, Tsinghua Shenzhen International Graduate School, Tsinghua University, Shenzhen 518055, China; Tsinghua-Berkeley Shenzhen Institute, Tsinghua Shenzhen International Graduate School, Tsinghua University, Shenzhen 518055, China; Institute of Materials Research, Tsinghua Shenzhen International Graduate School, Tsinghua University, Shenzhen 518055, China; Tsinghua-Berkeley Shenzhen Institute, Tsinghua Shenzhen International Graduate School, Tsinghua University, Shenzhen 518055, China; Institute of Materials Research, Tsinghua Shenzhen International Graduate School, Tsinghua University, Shenzhen 518055, China; Institute of Materials Research, Tsinghua Shenzhen International Graduate School, Tsinghua University, Shenzhen 518055, China; Institute of Materials Research, Tsinghua Shenzhen International Graduate School, Tsinghua University, Shenzhen 518055, China; Tsinghua-Berkeley Shenzhen Institute, Tsinghua Shenzhen International Graduate School, Tsinghua University, Shenzhen 518055, China; Institute of Materials Research, Tsinghua Shenzhen International Graduate School, Tsinghua University, Shenzhen 518055, China; Shenzhen Key Laboratory of Energy Materials for Carbon Neutrality, Institute of Technology for Carbon Neutrality, Shenzhen Institute of Advanced Technology, Chinese Academy of Sciences, Shenzhen 518055, China; Faculty of Materials Science and Energy Engineering, Shenzhen Institute of Advanced Technology, Shenzhen 518055, China; Shenyang National Laboratory for Materials Science, Institute of Metal Research, Chinese Academy of Sciences, Shenyang 110016, China

**Keywords:** polyamine adlayers, two-dimensional, graphene oxide, per- and polyfluoroalkyl substances, adsorption efficiency

## Abstract

The environmental occurrence of anthropogenic chemicals—especially persistent micropollutants of per- and polyfluoroalkyl substances (PFAS)—raises pressing concerns for global drinking-water safety. Adsorption is an effective technology for removing PFAS but is limited by unsatisfactory adsorption capacity and efficiency. We report a strategy to attach polyamine adlayers to graphene oxide (GO) nanosheets that produces highly charged and monodispersed 2D adsorbents of a GO nanosheet sandwiched between two 1-nm-thick polyamine adlayers. This adsorbent has a high adsorption capacity for PFAS of ∼3070 mg/g—tens of times greater than that of GO and commercial activated carbon. It also provides almost instant adsorption of a variety of PFAS and reaches 57%–95% of its equilibrium capacity in a minute and removes ∼100% of PFAS from contaminated water sources within a few minutes, transforming real-life PFAS-contaminated water into safe drinking water. Experiment and theory show that the planar nature of the 2D adsorbent combined with its abundant surface adsorption sites that electrostatically attract the polar groups of the PFAS, and hydrogen bonding and hydrophobic–hydrophobic interactions with their non-polar groups, account for its ultra-high adsorption capacity and rapid removal efficiency. We also show that regeneration of the adsorbents removes the adsorbed PFAS and allows subsequent destruction, demonstrating a closed-loop treatment solution for micropollutant contamination.

## Teaser

By sandwiching graphene oxide (GO) with two 1-nm-thin polyamine adlayers, GO, originally incapable of removing the persistent per- and polyfluoroalkyl substances from drinking water, is transformed into a highly efficient adsorbent

## INTRODUCTION

The safety of drinking water is a critical challenge worldwide [1,2]. One important reason is the prevalence of anthropogenic chemicals that are harmful to the environment and human health. They are difficult to remove but are increasingly discharged into groundwater and municipal water streams [3–5]. Per- and polyfluoroalkyl substances (PFAS) are one category of these chemicals. They contain multiple C–F bonds and those most frequently used are perfluorooctanoic acid (PFOA) and perfluorooctane sulfonate (PFOS) [1], which are utilized in many industrial processes and consumer products. These micropollutants are extremely hard to degrade, cause bioaccumulation and have an adverse effect on human health, including reproductive toxicity, high fetal and neonatal mortality, and a few cancers [6–9]. The removal of PFAS is, therefore, of great interest and at present achieved by using direct degradation, filtration, adsorption, plasma treatment and oxidation [2,10–13]. Among these methods, adsorption is considered to be the most effective technique, with a high efficiency, low cost and simplicity to integrate into industrial water-processing techniques [14–16].

An efficient adsorbent is the core of the adsorption process. Conventionally used adsorbents, such as granular activated carbon (GAC), only have a limited capacity for PFAS removal [17]. Recent advances have shown that some minerals, metal oxides, polymers, framework materials and nanocarbon materials such as carbon nanotubes can be used for PFAS removal [11,14,18–20] but they either have a small adsorption capacity at environmentally relevant concentrations (<1 mg/L) or require a long time (hours to days) to achieve adsorption equilibrium [19,21,22]. Therefore, the development of adsorbents with a high adsorption capacity and rapid/instant adsorption of PFAS, especially under an environment-related setting, is challenging.

Monolayer 2D materials have the ultimate thinness and their two sides are available as adsorption sites. Many 2D materials can now be mass-produced, so they are likewise of interest for efficient adsorbents. However, exploration of the use them as adsorbents in an aqueous phase, especially for the removal of PFAS, is limited and existing studies show unsatisfactory performance [23,24]. This is possibly because the existing 2D materials either have a weak surface charge and lack of a specific functional group that can generate strong interactions with polar and non-polar groups of PFAS and/or they suffer from poor dispersibility and stability in an aqueous environment, offsetting their intrinsic large surface area. Development of a 2D material that has good dispersibility that does not sacrifice the surface area and both sides that can strongly interact with the molecular chain of PFAS should enable efficient PFAS adsorption but hads yet to be achieved.

Polyethylenimine is a typical polyamine (PA) that is water-soluble and has a high density of amine groups [25], which can generate multiple interactions with the functional groups of 2D nanosheets (e.g. covalent bonds) and PFAS molecules (e.g. electrostatic attraction and hydrogen bonds). Therefore, that formation of a PA layer on the 2D nanosheets should offer a solution to the use of 2D materials for efficient PFAS adsorption. However, previous attempts have suggested notable agglomeration when the PA and 2D materials are mixed, mostly in graphene oxide (GO) nanosheets, resulting in an unsatisfactory adsorption capacity [26,27].

In this work, we report an ‘adlayer strategy’ to install a PA layer on both sides of a GO nanosheet, producing a sandwich 2D adsorbent with a thickness of ∼3 nm; this material is referred to as the PAGO. The PAGO shows a monodispersibility in water and the PA adlayer strongly adsorbs PFAS through various intermolecular forces. As a result, the PAGO is found to have an ultra-high adsorption capacity for PFAS and almost instant adsorption to remove PFAS in minutes. The adlayer-engineered PAGO provides an efficient way for PFAS removal that addresses drinking-water safety and opens up an enabling route for the design of 2D adsorbents.

## RESULTS AND DISCUSSION

### The formation of 1-nm-thick PA adlayers on a GO surface

**Figure 1.**
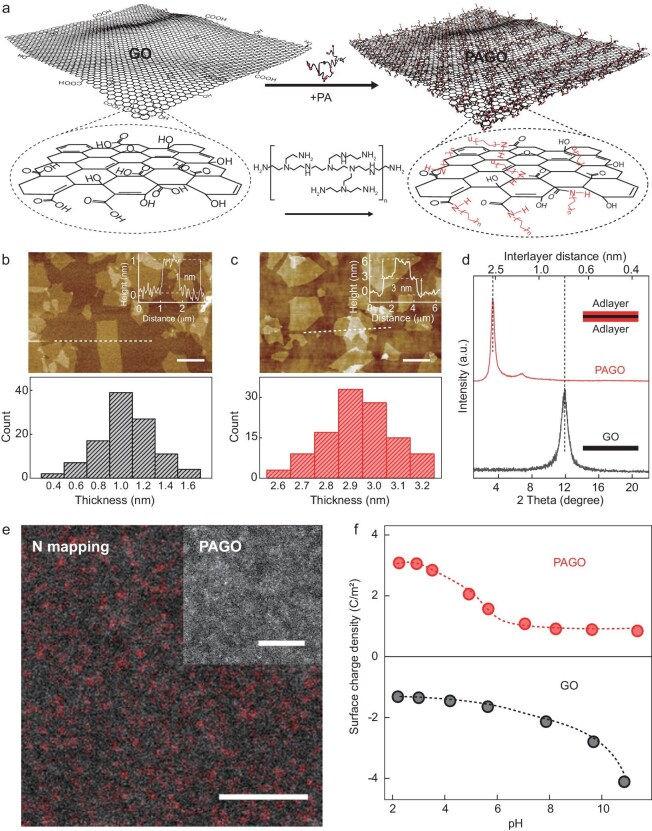
Preparation and structure of the PAGO. (a) Schematic of the preparation of PAGO nanosheets. (b, c) Upper panels are AFM images of (b) GO and (c) PAGO. Scale bars of (b) and (c) are 1 and 2 μm, respectively. Inset in the upper panels are the height profiles of the corresponding nanosheets measured along the white dashed lines. The lower panels are the thickness statistics of the corresponding GO and PAGO measured from >100 nanosheets. (d) XRD patterns of GO and PAGO laminates. (e) STEM image (inset) and corresponding nitrogen elemental map of a PAGO nanosheet. Scale bar of (e), 20 nm; scale bar of the inset, 10 nm. (f) Surface-charge densities of GO and PAGO at different pH values. The dashed curves are guides to the eye.

The PAGO was prepared by adding a commercial GO dispersion drop-wise into a polyethylenimine solution at a pH of ∼2 (Fig. 1a and Methods), during which the polyamine (PA) molecules became rapidly attached to the GO surface. This was evident from the fact that the surface charge of the GO nanosheets changed from negative (zeta potential ∼−50 mV) to positive (zeta potential ∼ +7.7 mV). After repeated washing of the PAGO with deionized water until the pH became neutral, the zeta potential increased to ∼ +43 mV and a stable PAGO colloidal dispersion was formed. There was no subsequent release of free PA from the PAGO into the dispersion, as confirmed by using ultraviolet–visible (UV–vis) spectroscopy (Section S1 and Fig. S1a) and its zeta potential remained above +40 mV for 7 days, suggesting good stability of the suspension (Table S1).

To probe the structure of the PAGO, we first used attenuated total reflectance-Fourier transform infrared spectroscopy (ATR-FTIR) and X-ray photoelectron spectroscopy. The results show the formation of amide functional groups between the PA and the GO (Section S1 and Fig. S2a and b), suggesting that the PAGO is a product in which the PA molecules are covalently grafted onto the GO nanosheets instead of being a simple mixture/physical attachment of the two. We then examined the thickness of the PAGO (>100 nanosheets were measured, Section S1 and Fig. S3) by using atomic force microscopy (AFM). It was found to be 2D with a thickness of ∼3 nm, which was a 2-nm thickness increase after the PA attachment (Fig. 1b and c). Both sides of the GO were covered by PA, which indicated that the PA adlayers were ∼1 nm thick. Such a structure was validated by using X-ray diffraction (XRD) analysis of a PAGO thin film. Compared with the original GO layer, the interlayer spacing of the PAGO increased by ∼2 nm (Fig. 1d). This means that, consistently with the AFM measurement, a 1-nm adlayer had formed on the GO nanosheets. Elemental mapping by using scanning transmission electron microscopy (STEM) provided information on the distribution of the PA molecules on the GO surface (Fig. 1e). A uniform distribution of N was observed whereas the original GO contained no nitrogen. All these results confirm that a uniform PA adlayer was formed on both sides of the GO nanosheets.

Because PA is a positively charged polyelectrolyte, an adlayer on the GO should change its surface charge, so we quantified the surface-charge density of the PAGO by using an acid–base titration technique that was reported previously [28,29]. We found that the PA adlayer altered the surface charge of the pristine GO from a negatively charged to a positively charged colloid GO, consistently with the zeta-potential measurements (Fig. 1f). Quantitatively, the surface-charge density of the PAGO was ∼1.08 C/m^2^ at a pH of ∼7 and increased to ∼3.1 C/m^2^ at a pH of ∼2 due to the increased protonation of the PA layer in an acidic solution. This charge density is significantly higher than those of reported 2D material nanosheets, including MoS_2_ and boron nitride, which are negatively charged nanosheets (note that, differently from the positively charged PAGO, they have a higher charge density at high pH but a lower charge density at a low pH; such a trend is dominated by the (de)protonation of, generally, oxygen-containing functional groups) and positively charged layer double hydroxides with charge densities that are in the range of 0.4–0.8 C/m^2^ [30–33]. It is worth noting that the charge of the 2D nanosheets is usually produced by the generation of edge and in-plane defects; the uniform charge distribution and ultra-high charge density of the PAGO indicate the advantage of our ‘adlayer’ strategy for non-destructive modulation of the surface properties of 2D materials.

### Ultra-high and instant adsorption of PFOA

**Figure 2.**
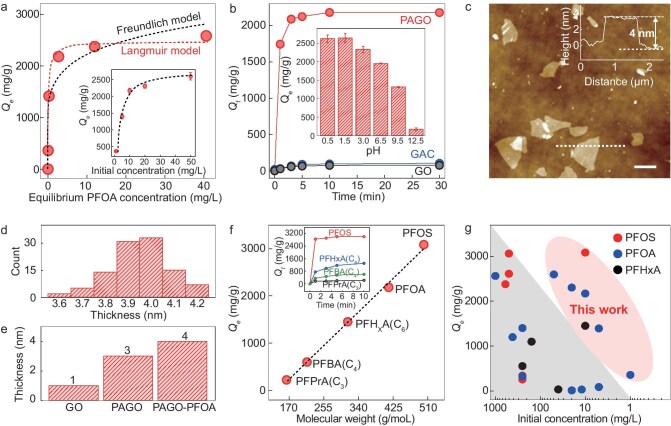
Efficient and rapid adsorption of PFAS by the PAGO. (a) Isotherm for the adsorption of PFOA by the PAGO. Inset are the *Q*_e_ values of PFOA by the PAGO at initial concentrations of 1–50 mg/L. The error bars are the standard deviations. (b) Effect of adsorption time on the adsorbed amount of PFOA on PAGO, GAC and GO measured by using the same protocol, and the initial PFOA concentration is 10 mg/L. Inset is the effect of the pH on the PFOA adsorption capacity of the PAGO and the error bars are the standard deviations. (c) AFM image of PAGO nanosheets after PFOA adsorption. Scale bar: 1 μm. Inset is the height profile of a nanosheet measured along the white dashed line. (d) Statistics of the thicknesses of PAGO nanosheets after PFOA adsorption. (e) Thicknesses of GO, PAGO and PAGO after PFOA adsorption. (f) Adsorption capacities of the PAGO for PFAS with different molecular weights. The dashed line is the best linear fit. Insets are the adsorption kinetics of PFOS, PFHxA, PFBA and PFPrA (10 mg/L) by the PAGO. (g) Comparison of PFOA, PFOS and PFHxA adsorption capacity when using the PAGO and other adsorbents. Symbols in the triangle area represent the adsorption capacity of the PAGO and symbols in the circle area are the previously reported data listed in Table S2.

The stable 2D PAGO nanosheets with a highly charged surface provide a high specific surface area and abundant adsorption sites that are essential in an efficient adsorbent. We then investigated its use as an adsorbent for the removal of emerging, persistent aqueous micropollutants. As shown in the inset of Fig. 2a, we studied the adsorption isotherm of the PAGO for PFOA, as an example, at various concentrations (room temperature, pH ∼ 7). It had an equilibrium adsorption capacity (*Q*_e_) of ∼2180 mg/g for a PFOA concentration of 10 mg/L and increases in the concentration to 50 and 100 mg/L produced an increase in and saturation of *Q*_e_ at ∼2600 mg/g. The ultra-high adsorption capacity that was observed at a PFOA concentration of 10 mg/L is an order of magnitude higher than that of the marketed GAC that is used in commercial Brita point-of-use filters (Brita AC,104.5 mg/g), pristine GO (75.2 mg/g) (Fig. 2b), and, to the best of our knowledge, is the highest among all previously reported adsorbents at a similar PFOA concentration (Fig. 2g) [14,16,34–36].

Further analysis of the adsorption isotherm shows a good fit to a Langmuir adsorption model (*R*^2^ for Langmuir model fitting and Freundlich model fitting are 0.99 and 0.91, respectively), indicating monolayer adsorption (Fig. 2a). To study this, we performed AFM measurements of the PAGO after its equilibrium adsorption of the PFOA (initial concentration: 100 mg/L). It was found that the PAGO retains a 2D structure with a thickness increase of ∼0.9–1 nm (Fig. 2c), i.e. a PFOA layer of thickness ∼0.45–0.5 nm is adsorbed on each side of the PAGO (Fig. 2d and e). This uniform thickness implies that the adsorbed PFOA is likely in the form of a packed monolayer. Note that the thickness increase is much smaller than the length of a PFOA molecule (∼1.1 nm, Section S2 and Fig. S5), suggesting that it is not adsorbed perpendicularly to the PAGO surface, but lies instead on the PAGO surface. If we assume that the PFAS molecules are aligned with each other, then this gives a simplified estimation that it sits at an angle of <30^o^ (arcsin (0.45/1.1) ≈ 24^o^) to the PAGO surface.

When measuring the adsorption capacity of the PAGO at different pH values from acidic to basic, we found the highest *Q*_e_ (∼2600 mg/g) at a pH of ∼1.5 (Fig. 2b, inset). Because PFOA has a chemically stable, charge-neutral C–F molecular chain, a negatively charged terminal carboxyl group and a pK_a_ as low as –0.5, this indicates that it exists in an anionic form in our tested pH range [37,38]. Therefore, differences in the observed dependence of *Q*_e_ on pH must be attributed to a change in the PAGO. A low pH promotes the protonation of the PAGO amine group, generating more electrostatic adsorption sites for the carboxyl group of PFOA, and hence a high *Q*_e_. A decrease in the pH to <1.5 produces no significant increase in *Q*_e_, possibly because the PAGO colloid becomes slightly unstable (zeta potential ∼38.21 mV). We note that, although a higher *Q*_e_ is observed in an acidic solution, because most PFAS exist in a pH-neutral aqueous environment, in this study, we focus on the adsorption of the PAGO at a pH of ∼7.

Not only does the PAGO have an ultra-high adsorption capacity for PFOA, but the adsorption is also very rapid. Its *Q*_t_ (the adsorption capacity at a certain time) reaches 82% and 100% of the *Q*_e_ in 1 and 10 min, respectively (Fig. 2b), suggesting almost instant removal. For example, it completely removes PFOA from water with a concentration of 10 mg/L in 5 min (Fig. S6a). We also tested the efficiency of removing PFOA with coexisting humic acid, which is a typical interfering substance that is used for PFAS adsorption (Fig. S6b) [14,21,39]. It was found that the PAGO was unaffected, with a ∼100% removal efficiency of PFOA (10 mg/L) after adsorption for a few minutes. Such rapid adsorption implies that most of the adsorption sites are available for PFOA adsorption, consistently with our observation of the ultra-high surface-charge density.

The ultra-high and rapid adsorption of PFOA by the PAGO extends to other PFAS species, including PFOS (perfluorooctane sulfonate), PFHxA (perfluorohexanoic acid), PFBA (perfluorobutanoic acid) and PFPrA (pentafluoropropionic acid). The PAGO has *Q*_e_ of ∼3071, 1389, 602 mg/g and 220 mg/g for PFOS, PFHxA, PFBA and PFPrA, respectively, which are, again, significantly higher than previously reported results at the same/similar concentrations (Fig. 2g and Table S2) [15,34]. It is known that many adsorbents are inefficient at removing short-chain PFAS [15] and, compared with the long-chain PFAS, the short-chain PFAS still exhibit high risks due to: (i) high hydrophilicity that enables a high solubility in water, which makes the phase-separation-based removal process difficult; (ii) enhanced mobility, which enables long-range transport via groundwater/surface water; (iii) extreme persistence (C–F bonds), which grants decadal-scale half-lives despite shorter chains; (iv) emerging toxicity, which disrupts endocrine/immune systems even at low doses; (v) rapid desorption from conventional adsorbents due to weak hydrophobic interactions. This synergy enables short-chain PFAS to bypass natural/engineered barriers, achieving global dispersion and retaining bioaccumulative hazards [10,15,18,34]. Our PAGO has effective adsorption of these species, promising its use for the remediation of water polluted that is by the most challenging short-chain PFAS.

### The adsorption mechanism

Comparison of the 2D PAGO with other porous adsorbents provides insight into its adsorption mechanism. Especially, previously reported adsorbents with amine groups, e.g. thick polyaniline nanotubes and GO/PEI agglomerates [26,40], when compared with the PAGO, showed limited adsorption sites to PFOA, so slower adsorption kinetics and a lower *Q*_e_ were reported. The observed rapid adsorption kinetics are attributed to the fact that the PAGO is 2D, with no intrinsic porosity, so that all the adsorption sites are exposed to the PFAS, avoiding intraparticle diffusion, which is a rate-limiting step for adsorption in many porous materials [41,42]. This is supported by a non-linear relationship between *Q*_t_ and the square root of the corresponding adsorption time, which is true for an intraparticle diffusion model (Section S2 and Fig. S7a). For the ultra-high adsorption capacity, it is known that the equilibrium adsorption capacity of porous adsorbents depends on the size and geometry of their nano-/mesopores, which provides spatial confinement/adsorption of the adsorbates [15,40]. Nevertheless, the PAGO inherits the non-porous nature of the GO, so the observed high *Q*_e_ should only depend on the surface adsorption, which is dictated by the abundant adsorption sites, and a strong interaction between the adsorbent and adsorbates, which eliminates desorption. The former is witnessed by the high surface-charge density and a good dispersibility of the PAGO (Fig. 1), whereas the latter has yet to be explored.

**Figure 3.**
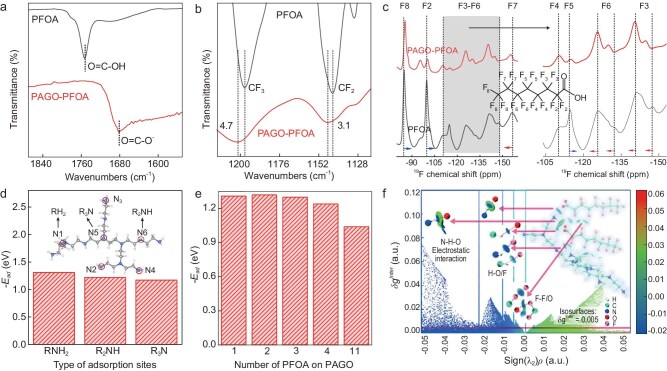
Elucidation of the adsorption mechanism. FTIR spectra of (a) carboxyl and (b) CF_3_ and CF_2_ functional groups of the PFOA and after its adsorption by the PAGO. (c) Solid-state ^19^F NMR of pristine PFOA and after its adsorption by the PAGO. The right panel shows the detailed NMR shifts of F3, F4, F5 and F6. (d) Calculated PFOA adsorption energy (–*E*_ad_) change along the type of amine sites and the number of adsorbed PFOAs on a PA unit. (e) Change in –*E*_ad_ with the number of PFOA molecules adsorbed. (f) Intermolecular interaction analysis by IGMH scatter graph ($\delta {g^{{\mathop{{inter}}} }}$ v.s. $sign({\lambda _2})\rho $) and the $\delta {g^{{\mathop{{inter}}} }}$ isosurfaces (the inset images). The scatter points and isosurfaces are plotted by the value of $sign({\lambda _2})\rho $ to analyse the type and strength of corresponded interactions, where $\delta {g^{{\mathop{{inter}}}}}$ is the difference between the magnitude sum of the fragment density gradients and the magnitude of the superposition of the fragment density gradients, and $sign({\lambda _2})\rho $ is the product of *ρ* (electron density) and the sign of *λ_2_* (the second largest eigenvalue of the Hessian matrix of *ρ*).

Therefore, we have performed further analysis to understand the interaction of PFAS with the PAGO. The pH-dependent adsorption behavior indicates that electrostatic adsorption between the negatively charged carboxyl or sulfonic acid groups of the PFAS and the amine group of the PAGO contributes significantly to the observed ultra-high *Q*_e_. Such electrostatic adsorption was confirmed by using FTIR analysis, which showed that the carboxyl group of the PFOA shifts from 1753 to ∼1681/cm after its adsorption on the PAGO (Fig. 3a), indicating the deprotonation of the carboxyl group that results from the electrostatic interaction between the PFOA and the PAGO [43,44].

Conversion of the *Q*_e_ values of different PFAS to their molar adsorption capacities (*Q*_e_*^m^* = *Q*_e_/molecular weight) indicates that other intermolecular forces also account for the observed PFAS adsorption. Specifically, if the charged end group of the PFAS is the only adsorption site for the PAGO, because the tested PFAS have only one negatively charged terminal group, *Q*_e_*^m^* should be independent of the C–F chain length. However, *Q*_e_*^m^* increases with the length of the C–F chain (Fig. 2f and Table S3). For example, the PAGO has a *Q*_e_*^m^* for PFOA that is 3.8 times higher than that of PFPrA, suggesting that the charge-neutral C–F chain is important for the observed ultra-high *Q*_e_. This is in good agreement with our AFM measurement (Fig. 2c), which shows that the adsorbed PFOA molecules tend to lie on the PAGO rather than normal to it, suggesting that charge-neutral molecular chains have a strong interaction with the PAGO, so the entire PFOA molecule is attracted/pulled close to its surface.

Indeed, our FTIR analysis (Fig. 3b) shows a blue shift of the C–F stretch band in the PFOA after adsorption (CF_2_ and CF_3_ of the PFOA molecule shift by 3.1 and 4.7 cm^−1^, respectively) [45]. Detailed FTIR analysis shows that the amine group of the PAGO blue shifts by 19.6 cm^−1^ (Fig. S7c) and ^19^F-NMR (nuclear magnetic resonance) analysis (Fig. 3c) shows an upshift of F2, F5 and F8 of the PFOA (as indicated in Fig. 3c, F1, F2…F8 are the positions of the fluorine atoms in a PFOA molecule). The collective evidence suggests the formation of hydrogen bonds between the C–F of the PFOA and the NH_2_ of the PAGO [46,47]. In addition, NMR analysis shows that F3, F6 and F7 of the PFOA downshift by 1–3 ppm, suggesting a hydrophobic–hydrophobic interaction between the intermolecular C–F bonds [14]. This intermolecular interaction between neighboring PFOA molecules is likely to guide the self-arrangement and result in packing of the adsorbed PFOA, which agrees with AFM analysis that uniform adsorption of PFOA is observed on the PAGO.

From all the above-mentioned evidence, we conclude that the electrostatic attraction between the carboxyl group of the PFOA and the amine group of the PAGO, the hydrogen bonds between the C–F chain of the PFOA and the amine group of the PAGO, as well as the F–pF hydrophobic–hydrophobic interaction between two packed PFOAs account for the adsorption interaction between the PFOA and the PAGO. In addition, because multiple positions of a PFOA molecule (the charged and charge-neutral groups) are involved in the adsorption process, it is reasonable to believe that such strong and multiple adsorption interactions inhibit the diffusion and desorption of the adsorbed PFOA on the PAGO surface, giving a high equilibrium adsorption capacity and rapid adsorption.

To gain quantitative insight into the adsorption mechanism, the experimentally observed adsorption interactions were examined by using theoretical calculations. Density functional theoretical (DFT) calculations were performed to investigate the adsorption energy of the PFOA on the PAGO (–*E*_ad_). Considering that there are three types of amine groups on the PAGO (primary, secondary and tertiary), the –*E*_ad_ values of the PFOA on different types of amine groups were calculated (Fig. 3d) to be ∼ –1.31, ∼ –1.22 and –1.17 eV for the primary, secondary and tertiary amine groups, respectively. Furthermore, to understand why the primary amine group has stronger adsorption to the PFOA, we calculated the –*E*_ad_ of the PFOA on the primary amine group of linear PA and found that the –*E*_ad_ was 1.2 eV, which is smaller than the branched one. Detailed analysis shows that such difference originates because that PFOA forms hydrogen bonds with the side chains of the branched polymer, and hence increase the –*E*_ad_, but such an interaction is difficult for linear chain polymers (Fig. S4g). We note that –*E*_ad_ is higher for the primary amine group than the other two types of amine groups, but such a difference in –*E*_ad_ is minor, i.e. all types of amine groups on the PAGO are energy-favorable adsorption sites for PFOA. Moreover, with a further increase in the number of PFOA molecules that are adsorbed on one PA unit, from 1 to 2, 3, 4, … and up to 11 (the maximum number of adsorbed PFOA molecules per PA unit considering the electrostatic interaction between the charged groups of the PFOA and the PA), there is only a negligible decrease in –*E*_ad_ (Fig. 3e and Fig. S8), suggesting that the adsorption of multiple PFOA molecules on one PA unit is energy-favorable, which explains the experimentally observed ultra-high and rapid adsorption (Fig. 2b).

We next identified the interaction between the PFOA and the PA by using modeling a process in which two PFOA molecules were step-by-step adsorbed onto one amine site (Section S2 and Fig. S8). The isosurface of the charge-density difference shows that the adsorption involves an interaction between the PFOA and the PA as well as an interaction of the C–F chains between two neighboring PFOA molecules (Section S2 and Fig. S8c) and is consistent with experimental analysis. To distinguish the contribution of the different interactions, we used an independent gradient model based on Hirshfeld partition (IGMH) function analysis (Fig. 3f). The results show that the electrostatic attraction and the hydrogen-bond interactions between the PFOA and the PA, as well as the fluorine–fluorine and fluorine–oxygen interactions between the two PFOA molecules, account for the PFOA adsorption. In addition, the intermolecular interaction analysis between the PFOA and the PA (Fig. 3f and Section S2) reveals that N–H–O electrostatic interaction exhibits the largest electron density ($sign({\lambda _2})\rho $ = ∼ −0.05 a.u.) and accounts for the strongest adsorption interactions, followed by hydrogen-bond interactions (H–O/F) and F–F/O interactions. Finally, we examined the adsorption interactions when 11 PFOA molecules were adsorbed on a PA unit (Section S2 and Fig. S8d and e) and again found that all interactions, including electrostatic attraction, hydrogen bonding and hydrophobic–hydrophobic bonds, account for the observed adsorption, confirming the experimental analysis.

### PFOA cleanup practice under environment-related settings and adsorption regeneration

**Figure 4.**
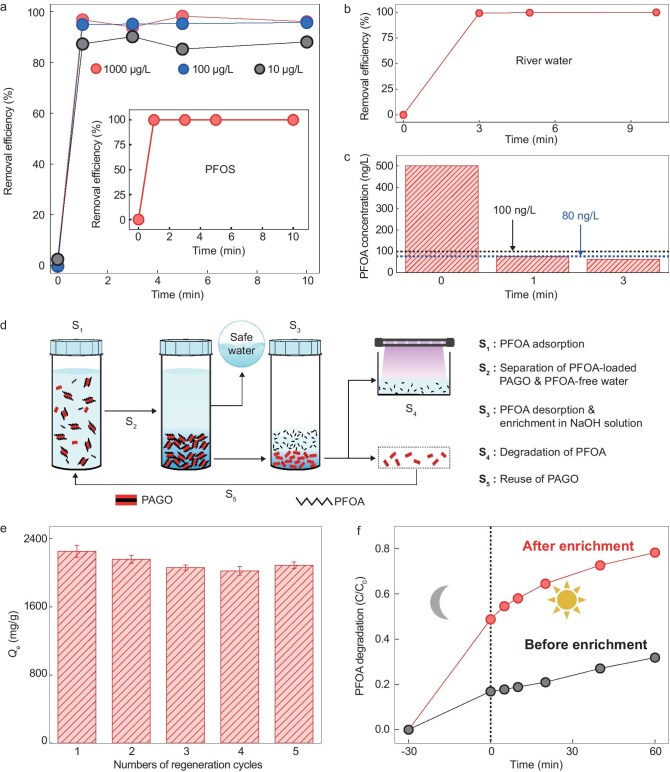
Removal of PFAS at environmental concentrations, the regeneration of the PAGO and the photocatalytic degradation of adsorbed PFAS. (a) Removal of PFOA by the PAGO at various environmental concentrations. Inset is the removal efficiency of PFOS (∼18 μg/L) by the PAGO. (b, c) Adsorption performance of the PAGO in removing PFOA from (b) contaminated river water and (c) simulated PFOA-contaminated tap water. (d) Illustration of desorption, enrichment and photocatalytic degradation of PFOA. (e) *Q*_e_ of PFOA removal by the PAGO regenerated through different numbers of cycles. (f) Photocatalytic degradation of PFOA before and after its concentration enrichment.

The ultra-high and rapid adsorption of PFAS by the PAGO suggests its use for the removal of PFAS at environmental concentrations. Most PFAS (mainly PFOA and PFOS) in water, including surface water, groundwater and drinking water, have concentrations of <1000 μg/L and the upper concentration limits of PFAS in drinking water that were set by the European Union (EU) and China are 100 and 80 ng/L, respectively [48,49]. We therefore first examined the removal of PFOA at a level of 10–1000 μg/L and observed ∼100% removal of PFOA with initial PFOA concentrations of 0.1 and 1 mg/L within 1 min. Even at a 10-μg/L concentration, after 1 minute of adsorption, the removal efficiency reached ∼94% (Fig. 4a).

We also examined the removal of PFOA from contaminated river water that was collected from the Xiaoqing River (∼160 μg/L, Zibo, China) and simulated PFOA-contaminated municipal tap water (∼500 ng/L). For the river water, we achieved a ∼100% removal efficiency in 1 min (Fig. 4b) and the PFOA concentration decreased by nearly three orders of magnitude after 10 min of adsorption, reaching 70 ng/L (Section S3 and Fig. S9). For the simulated PFOA-contaminated municipal tap water, the concentration decreased from 500 to 76 ng/L in 1 min and to ∼61 ng/L in 3 min, meeting the drinking-water standards of both the EU (100 ng/L) and China (80 ng/L) (Fig. 4c). Thus, the PAGO can efficiently transform contaminated water into safe, drinkable water, indicating its use for practical water treatment. Considering the integration of the PAGO into a conventional water-treatment process, we have tried the use of the PAGO in a fixed-bed column for continuous PFOA adsorption. As a demonstration, we coated the PAGO on cotton (the sample was noted as PAGO-coated cotton), which is a widely used material for fixed-bed column water treatment [50,51]. The PAGO was found to be uniformly coated on individual cotton fibers. Although the pristine cotton showed no removal efficiency (∼0%) throughout the experiment, the PAGO-coated cotton had >99.38% removal efficiency for 86 L of PFOA solution (Section S3 and Fig. S10), suggesting that the PAGO is promising for practical continuous PFAS removal.

### Adsorption regeneration and PFOA degradation

Although the PAGO removes PFAS from water and converts it into safe, drinkable water, the PFAS remains in the solid or liquid waste stream after adsorption, which still poses environmental and health risks. This involves two steps: as shown in Fig. 4d, the first is desorption of the PFOA from the PAGO and the second is photocatalytic degradation of the desorbed PFOA by using a well-established catalyst: In_2_O_3_ [52].

For the PFOA desorption and regeneration of the PAGO, we found that simply soaking the adsorbents in a NaOH solution (2 M for 1 h) efficiently stripped the adsorbed PFOA from the PAGO and regenerated the PAGO. This can be seen from the fact that, even after several repeated regenerations, the PAGO had a *Q*_e_ value of >2000 mg/g (Fig. 4e). We believe that the effectiveness of NaOH for desorption is possibly because of its deprotonation of the PAGO and a neutralization between the acidic PFOA and the NaOH, as previously reported [53].

The second step is the photodegradation of the desorbed PFOA. Initially, after the adsorption of 0.5 mg/L PFOA solution (we chose this low concentration because it is within the environmentally relevant concentration range) by the PAGO, the PFOA was desorbed and we obtained a PFOA solution with the same concentration of 0.5 mg/L (see Methods). In this case, we found that the photodegradation efficiency was low (∼37% within 60 min). This is not surprising, as it has been reported that the degradation efficiency decreases at low PFOA concentrations, possibly because mass transportation becomes the rate-limiting step at low concentrations [54,55]. We then measured the influence of the PFOA concentration on its photocatalytic degradation efficiency. It was found that the photodegradation efficiency increased to ∼80%–90% when the PFOA concentration was increased to 5–10 mg/mL (Section S4 and Fig. S11). This indicates that a higher concentration of PFOA is favored for its degradation. Fortunately, in our case, a high PFOA concentration could be achieved simply by controlling the volume of NaOH solution that was used for the PFOA desorption. For example, after the adsorption of 100 mL of 0.5 mg/L PFOA solution, the PFOA-adsorbed PAGO was regenerated in a 10-mL NaOH solution; with a ∼100% removal efficiency, the desorbed PFOA concentration in the NaOH solution increased to 5 mg/L and the photodegradation efficiency increased to ∼78.3% (after 60 min of degradation) (Fig. 4f). Together, combining high removal efficiency and the photocatalytic degradation of adsorbed PFOA, we demonstrate not only the removal, but also the detoxification, of the PFAS.

## SUMMARY

We have reported an adlayer strategy to build a new type of 2D material that consists of a GO nanosheet that is sandwiched between two polyamine layers. The resulting PAGO nanosheets are mono-dispersible in water and have an ultra-high surface positive charge and strong interactions with both the polar and non-polar groups of PFAS. Such properties give a record-high and almost instant adsorption of PFAS from water. When tested at an environment-relevant concentration, the PAGO transformed PFAS-contaminated water into safe, drinkable water that met EU and China standards after only a few minutes. We also used an ‘adsorption-enrichment-photocatalytic degradation’ process to not only efficiently remove, but also degrade, the PFAS. Our adlayer strategy opens up a new way to design 2D materials at room temperature with new functionalities. The demonstrated ultra-high and rapid removal of PFAS by the PAGO provides a usable solution to PFAS contamination. Considering the complexity of contaminants and their minute concentrations in water, the adlayer material could be extended to molecules other than polyamine; this would effectively alter the surface properties of 2D materials, as interparticle diffusion is eliminated for 2D adsorbents, which have a high surface area, and the resulting adlayer-engineered 2D materials, though without porosity, could be used as high-performance adsorbents to tackle global water contamination problems.

## METHODS

All reagents were purchased from Macklin Biochemical and Sigma-Aldrich and used as received unless otherwise stated.

### Preparation of PAGO

Commercially available GO was purchased from Shenzhen Matterene Technology. Colloidal PAGO dispersions were prepared by mixing 10 mL of 0.3 mg/mL solution of GO in ultrapure water at a pH of ∼6 and 20 mL of an 18 mg/mL solution of PA (branched PA, average Mw ∼ 25k and Mn ∼ 10k) in a 0.1 M NaCl solution (pH ∼ 2, adjusted by adding 1 M HCl solution). The mixture was mixed for 1 h by using a Vortex Mixer (700 r/min) and was then centrifuged at 11 000 r/min for 30 min. The sediment was collected and repeatedly washed with ultrapure water. The process was repeated five times until no PA was released into the water, as determined by using UV–vis spectrophotometry (Section S1 and Fig. S1a), and the sediment was collected and used as the PAGO adsorbent. Using the *Q*_e_ to 10 mg/L PFOA as a reference, we also studied the influence of different types of PA on the effectiveness for PAGO synthesis. PA with Mw ∼ 70k and Mw ∼ 1.8k can form the PAGO but with an average thickness of ∼2.6 nm because of incomplete coverage of the PA on the GO surface, and the *Q*_e_ values are ∼1780 and 1665 mg/g, respectively. A PA with a low Mw of 600 and 800 formed agglomerates with the GO when the identical preparation procedure was used, resulting in a low *Q*_e_ (Fig. S4f). We therefore used the branched PA with Mw ∼ 25k for the preparation of the PAGO.

### Regeneration of the PAGO with adsorbed PFOA

After adsorption, the PFOA-adsorbed PAGO was regenerated by soaking it in a NaOH solution (1 M, 50 mL) and shaking for 2 h. The mixtures were centrifuged at 11 000 r/min for 30 min and the sediments were collected and washed five times with ultrapure water until the pH of the supernatant became neutral. The sediment that was the regenerated PAGO was used for the next adsorption cycle.

### PFOA desorption, enrichment and photocatalytic degradation

The procedure involved three steps: (i) desorption—following the same regeneration procedure, the PAGO (0.5 mg/mL, 14 mL) with adsorbed PFOA (2.1 L, 500 μg/L) was soaked in 175 mL of a 2 M NaOH solution for 1 h; (ii) separation—the mixture was then centrifuged and the supernatant containing the desorbed PFOA was neutralized with HCl, resulting in 200 mL of PFOA solution with a concentration of ∼5 mg/L for use in photocatalytic degradation; it should be noted that ∼5% of the PFOA was lost during this process due to unavoidable sample waste when taking the supernatant collection. The sediment was collected as regenerated PAGO for further adsorption experiments; (iii) photodegradation—before UV irradiation, 10 mg of In_2_O_3_ (50 mg/L) was added to the PFOA solution and stirred for 30 min in the dark to ensure adsorption–desorption equilibrium. The photodegradation experiments were conducted in a quartz tube with a 10-W, 254-nm UV lamp with an average irradiation intensity of ∼35 W/m^2^, and the suspensions were sampled at given intervals to determine the PFOA concentration, which was measured by using the same procedure as was used for the adsorption experiments.

## Supplementary Material

nwaf092_Supplemental_File

## Data Availability

The data supporting the findings in this study are available within the paper and its Supplementary information. Source data are provided in this paper.
