# Supplementary material for: Graphene oxide with 1-nm-thick adlayer for efficient and near-instant removal of per- and polyfluoroalkyl substances
Source: Natl Sci Rev. 2025 Mar 7;12(5):nwaf092. doi: 10.1093/nsr/nwaf092 (PMC11974386; doi:10.1093/nsr/nwaf092)
Supplement: nwaf092_Supplemental_File [file nwaf092_supplemental_file.pdf]

Graphene Oxide with 1-nm-thick Adlayer for Efficient and Near-instant Removal of Per- and Poly-fluoroalkyl Substances

Dingxin Xu[1,#], Wenhui Ding[2,#], Xinyu Gong[1], Xianjun Tan[2], Hang Li[1], Fei Li[1], Mingrui Zhang[1], Yuxiong Huang[2,*], Yang Su[1,*], Hui-Ming Cheng[3,4,5,*]

1 . Institute of Materials Research, Tsinghua Shenzhen International Graduate School, Tsinghua University, Shenzhen 518055, China;

2 . Tsinghua-Berkeley Shenzhen Institute, Tsinghua Shenzhen International Graduate School, Tsinghua University, Shenzhen 518055, China;

3 . Shenzhen Key Laboratory of Energy Materials for Carbon Neutrality, Institute of Technology for Carbon Neutrality, Shenzhen Institute of Advanced Technology, Chinese Academy of Sciences, Shenzhen 518055, China;

4 . Faculty of Materials Science and Energy Engineering, Shenzhen Institute of Advanced Technology, Shenzhen 518055, China;

5 . Shenyang National Laboratory for Materials Science, Institute of Metal Research, Chinese Academy of Sciences, Shenyang 110016, China

*__Corresponding authors__. E-mails: huang_yuxiong@sz.tsinghua.edu.cn; su.yang@sz.tsinghua.edu.cn; hm.cheng@siat.ac.cn

#Equally contributed to this work.

Supplementary Section 1 Preparation and characterization of a polyamine adlayer on graphene oxide (PAGO) nanosheet

1.1 Preparation of PAGO

During the synthesis of PAGO, because of the electrical charge attraction, when the Graphene Oxide (GO) dispersion is added to the polyamine (PA), the GO agglomerates, which is evident from its very low zeta potential (7.7 mV, Table S1). However, such agglomerates are weakly held together as “soft agglomerates” in which the excess PA is weakly attached (for example, by van der Waals interaction) to the GO. To test this, we have repeatedly washed the material with ultrapure water followed by centrifugation (11000 r/min for 30 minutes). After each centrifugation, the sediment was collected and re-dispersed in water for the next cycle. During the process, the supernatant was collected and examined by Ultraviolet-visible (UV-Vis) spectroscopy to determine the excess PA molecules in it. We find that after 5 washing cycles, the adsorption peak of PA becomes negligible, indicating that all the excess/free PA molecules are removed (Fig. S1a). Furthermore, the zeta potential of the final PAGO is 42.9 mV, suggesting a stable and positive charge of the colloid. We also monitored the colloidal stability over time and found that the zeta potential remains at ~41 mV after 7 days, suggesting its long-term stability (Table S1).

Table S1 Zeta potentials of GO, and PAGO during and after the preparation process

Materials	Zeta (mV)
GO	-49.7
PAGO after water wash	42.9
After 7 days	41

Because the preparation of PAGO involves the mixing of two components (GO and PA), we changed the initial mixing ratio of PA and GO and studied its influence on the PA’s mass loading on the resulting PAGO. Specifically, a certain amount of the GO dispersion was added to PA solutions, for example, in our case, 10 mL of 0.3 mg/mL GO dispersion was added dropwise to 20 mL of 0.15, 4.5, 12, 18 and 30 mg/mL PA solutions to achieve initial mixing ratios (PA/GO) of 1, 30, 80 120 and 200, respectively. The mixtures were then washed using an identical procedure to that mentioned above and dried at 60 ℃ for 12 h. The weight difference between the

original GO and the obtained PAGO (m_{PAGO}) was the mass loading of PA (m_{PA}) on the PAGO, and its weight percentage (PA%) was calculated as 100%xm_{PA}/m_{PAGO}. As shown in Fig. S1b, when the initial weight ratio of PA to GO increases from 0 to 200, the weight percentage of PA on the PAGO increases monotonically and reaches a plateau at 61.1% when the initial weight ratio reaches 120. We have also measured the adsorption capacity (Q_e) of PFAS by PAGO with different PA loadings (10 mg/L perfluorooctanoic acid (PFOA) and 1 h adsorption at room temperature). As shown in Fig. S1c, The Q_e increases with the PA loadings, and saturated at ~61.1% PA loading (Fig. 1c), the highest PA loading we can achieve. Therefore, we used the mass ratio (PA/GO) of 120 for the study.

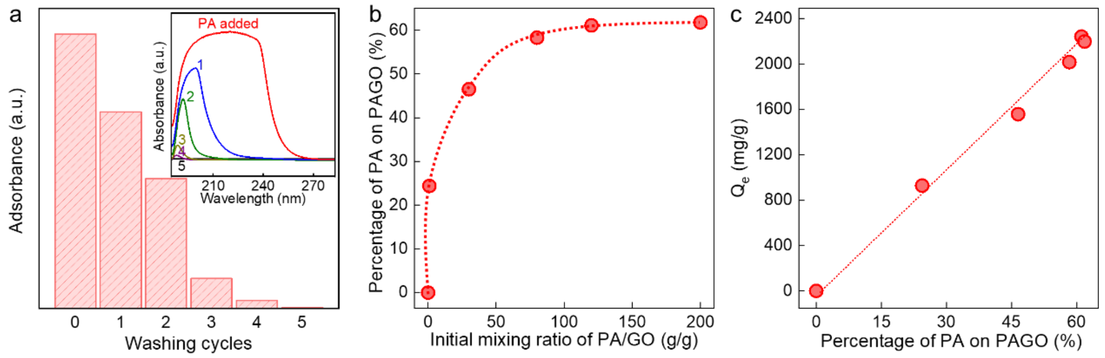

Fig. S1. Control of the PA content in the resulting PAGO hybrid nanosheets. (a) The change of absorbance of free PA released into the supernatant for different washing cycles. Inset is the UV-Vis spectra of PA in the supernatant after each cycle (the number of cycles is indicated on the corresponding spectrum). (b) Weight percentages of PA on PAGO prepared with various initial PA/GO mixing ratios. (c) The Q_e of PFOA by PAGO with different PA loadings.

1.2 Material Characterization of PAGO

ATR-FTIR (attenuated total reflectance-Fourier transform infrared spectroscopy) was used to study the interaction between PA and GO (Fig. S2a). Compared to the original GO, the spectrum of the PAGO sample shows that, the N-H bands of primary and secondary amides appear at 1650-1580 cm^{-1} and 1500-1530 cm^{-1}, and the C-N bands of primary and secondary amides appear at 1420-1400 cm^{-1} and 1300-1260 cm^{-1}, suggesting the successful addition of PA to GO nanosheets. More importantly, the 1670-1630 cm^{-1} band belonging to the C=O stretching vibration of tertiary amides, indicates the possible covalent attachment of PA to the GO, resulting in the conversion of amine groups into stable amide groups[1]. The formation of amide groups was validated by X-ray photoelectron spectroscopy (XPS). In Fig. S2b, compared to

the original GO, the prominent peak at 286.0 eV is attributed to C-N, indicating a covalent bonding between GO and PA[2].

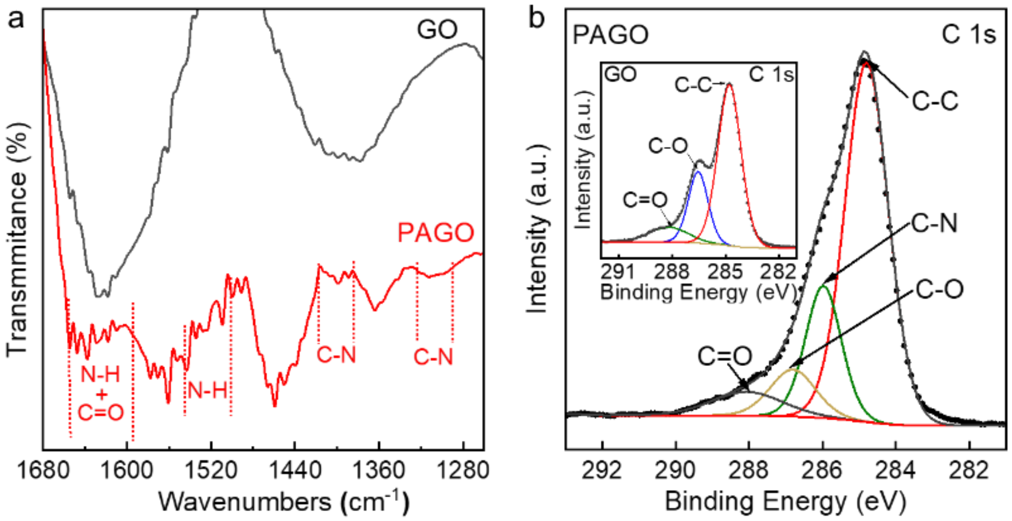

Fig. S2. FTIR and XPS analysis of GO and PAGO. (a) ATR-FTIR spectra of GO and PAGO. (b) XPS spectra of C 1s in the GO (inset) and PAGO.

To measure the thicknesses of GO, PAGO, and PAGO with adsorbed PFOA, >100 nanosheets of each sample were measured by atomic force microscopy (AFM). Fig. S3 shows the AFM images used for obtaining the thickness statistics.

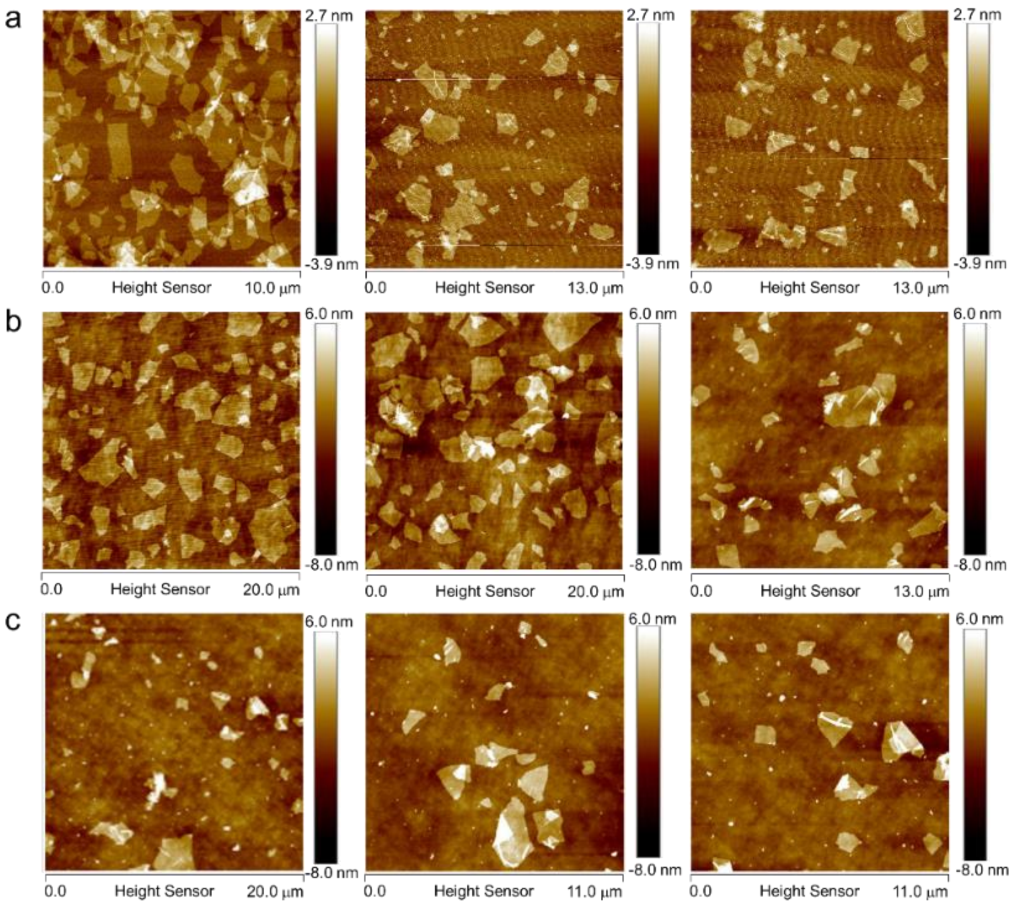

Fig. S3. AFM images of nanosheets of (a) GO, (b) PAGO and (c) PAGO with

adsorbed PFOA.

1.3 Estimation of surface charge density

The surface charge densities of GO and PAGO nanosheets were estimated using an acid-base titration technique[3]. Prior to the titrations, the dispersions as well as the background aqueous solution were stirred for 1 hour. Titrations started either from a low pH (GO aqueous dispersion, 0.5 mg/mL), or high pH (PAGO aqueous dispersion, 0.5 mg/mL) using 0.1 M NaOH or 0.1 M HCl as base/acid titrants. Both dispersions were titrated in the pH range from ~2 to 11. The same procedure was performed for the ultrapure water without the presence of GO or PAGO flakes. The surface charge (σ_s) of GO and PAGO was calculated using Eq. (1):

$$\sigma_S = F.\left(-\frac{C_{\frac{NaOH}{HCl}}(V_b - V_d)}{s.\gamma.V}\right) \qquad (1)$$

where F is the Faraday constant, C_{NaOH} and C_{HCl} are the concentrations of the titrants (mol/L), V_b is the volume of the titrant added in a blank titration which would correspond to the volume V_d of titrant added in the titration of the dispersion (L) to reach the same pH, and s is the specific surface area of the solid (surface area divided by the mass of solid particles), and γ is the mass concentration of the solid (mass of solid divided by the volume of the total liquid medium V).

Supplementary Section 2. Analysis of PAGO's adsorption of per- and poly-fluoroalkyl substances (PFAS)

2.1 Adsorption methods

Isotherm for the adsorption of PFAS by PAGO

Typically, a small amount of the PAGO dispersion (0.2 mL, 0.5 mg/mL) was added to polypropylene bottles containing PFOA solutions (30 mL) with different concentrations (pH~7). The mixtures were stirred (400 r/min) at room temperature for 30 minutes, then filtered through a membrane filter (pore size ~0.22 μm). The concentrations of PFOA in the filtrates were analyzed using high-performance liquid chromatography (HPLC). Specifically, for PFOA, perfluorooctane sulfonate (PFOS), perfluorohexanoic acid (PFHxA), Perfluorobutanoic acid (PFBA) and pentafluoropropionic acid (PFPrA) solutions with concentrations of 1-100 mg/L, 1-1000 μg/L, and 10 ng/L-1 μg/L, their concentrations were measured by HPLC,

liquid chromatography with a triple quadrupole mass spectrometer (LC-MS/MS) and ultra-high-performance liquid chromatography respectively (UHPLC). The adsorption capacity, Q_e (mg/g) was calculated using Eq. (2):

$$Q_e = \frac{(C_0 - C_e) \times V}{m} \quad (2)$$

where C_0 is the initial concentration of PFOA (mg/L), C_e is the equilibrium concentration in the filtrate, V is the volume of the PFOA solution (L) and m is the mass of PAGO (g). The experiments were repeated for three different samples. For the Langmuir model, Eq. (3) was used.

$$Q_e = \frac{Q_{max} b C_e}{1 + b C_e} \quad (3)$$

where Q_{max} (mg/g) is the maximum capacity of the adsorbate required to form a complete monolayer on the surface and b is the Langmuir constant. For the Freundlich model, Eq. (4) was used.

$$Q_e = K_F C_e^{1/n} \quad (4)$$

where K_F is the Freundlich constant and is related to the adsorption capacity of the material, and $1/n$ is a constant related to surface heterogeneity.

<u>Study of adsorption kinetics</u>

A 0.2 mL, 0.5 mg/mL PAGO dispersion was added to 30 mL of PFAS including PFOA, PFOS, PFHxA, PFBA, and PFPrA solutions (10 mg/L for each PFAS solution) under continuous stirring. The mixture was collected at different times (0, 1 minute, 3 minutes, 5 minutes, and 10 minutes) and filtered through a membrane filter (pore size ~0.22 μm). The PFAS concentrations in the filtrates were analyzed by HPLC, LC-MS/MS, and UHPLC. The amounts of PFAS adsorbed by PAGO at different times (Q_t) were determined by Eq. (5):

$$Q_t = \frac{(C_0 - C_t) \times V}{m} \quad (5)$$

where C_t is the concentration of PFOA in the filtrate at a given adsorption time, and Q_t is the adsorption capacity after different adsorption times. To measure the influence of pH on Q_t, the same procedures were followed but with the pH (0.5, 1.5, 3.0, 6.5, 9.5, and 12.5) adjusted by adding either HCl or NaOH.

<u>PFAS removal from environmental concentrations</u>

0.2 mL (0.5 mg/mL) of PAGO dispersion was added to 30 mL of a PFOA/PFOS solution (initial concentration ranges from 10 μg/L to 1000 μg/L). After 10 minutes adsorption, the mixtures were filtered and the concentration of PFOA/PFOS was

analyzed by LC-MS/MS and UHPLC to determine the removal efficiency (R) using Eq. (6).

$$R = \frac{C_0 - C_t}{C_0} * 100\% \qquad (6)$$

PFOA-contaminated river water was collected from the Xiaoqing River in Zibo China. The simulated PFOA-contaminated municipal water is made by adding PFOA to the tap water of Shenzhen city.

2.2 Adsorption Capacity Comparison

To investigate how different molecular weights (M_W) and structures of PAs affect the synthesis of the resulting PAGO nanosheets and their Q_e, we employed the identical fabrication procedure but only changed the branched PA with a M_W of 25k to the linear PA with a M_W of 70k, 1.8k, 600 and branched PA with a M_W of 800. The resulting samples were denoted as L70k-PAGO, L1.8k-PAGO, L600-PAGO and B800-PAGO.

The L70k-PAGO and L1.8k-PAGO both can form stable colloids. The average thicknesses of both samples are ~2.5 nm, ~0.5 nm thinner than PAGO because of incomplete coverage of PA on the GO surface (Fig. S4a-e). The corresponding Q_e (initial PFOA concentration 10 mg/L) are ~1780 and ~1665 mg/g respectively. These values decreased by ~16% and 21.6% compared to the PAGO (Fig. S4f). The low Mw of linear PA (600) and branched PA (800), which have very low coverage on the GO and form an unstable agglomerate, result in low Q_e.

To explain why linear PEI has low coverage on the GO surface, we recall that one of the major structural differences between linear and branched PEI is that the linear PEI only has two primary amine groups at both ends with the rest being secondary amine groups, but the branched PEI has approximately 25% primary amine group. The primary amine group is known to have a stronger ability to react with the carboxyl group on the GO surface because of a higher degree of protonation[4,5], forming the covalent amide group which anchors the PEI on the GO surface. Because of a small fraction of the primary amine group, compared to the branched PEI, the linear PEI should generate less amide group with GO, therefore, has a weaker interaction with GO, explaining a lower coverage and a smaller thickness found for L-PAGO.

Not only does linear PEI have a lower coverage on the PAGO that decreases the

Q_e, but we compared the adsorption energies (-E_{ad}) of PFOA on the different amine groups of linear and branched PA (VASP-5.4.4, Fig. S4g). All calculation conditions are the same with that of the -E_{ad} calculated for PAGO described in the Supplementary Section 5. The simulation shows that, for primary amine group on the B-PAGO, it has higher PFOA -E_{ad} than that on the L-PAGO. This is because the adsorbed PFOA can form extra hydrogen bonds with the side chain of B-PAGO, but this would be difficult for the PEI with linear chain structure (Fig. S4h-i).

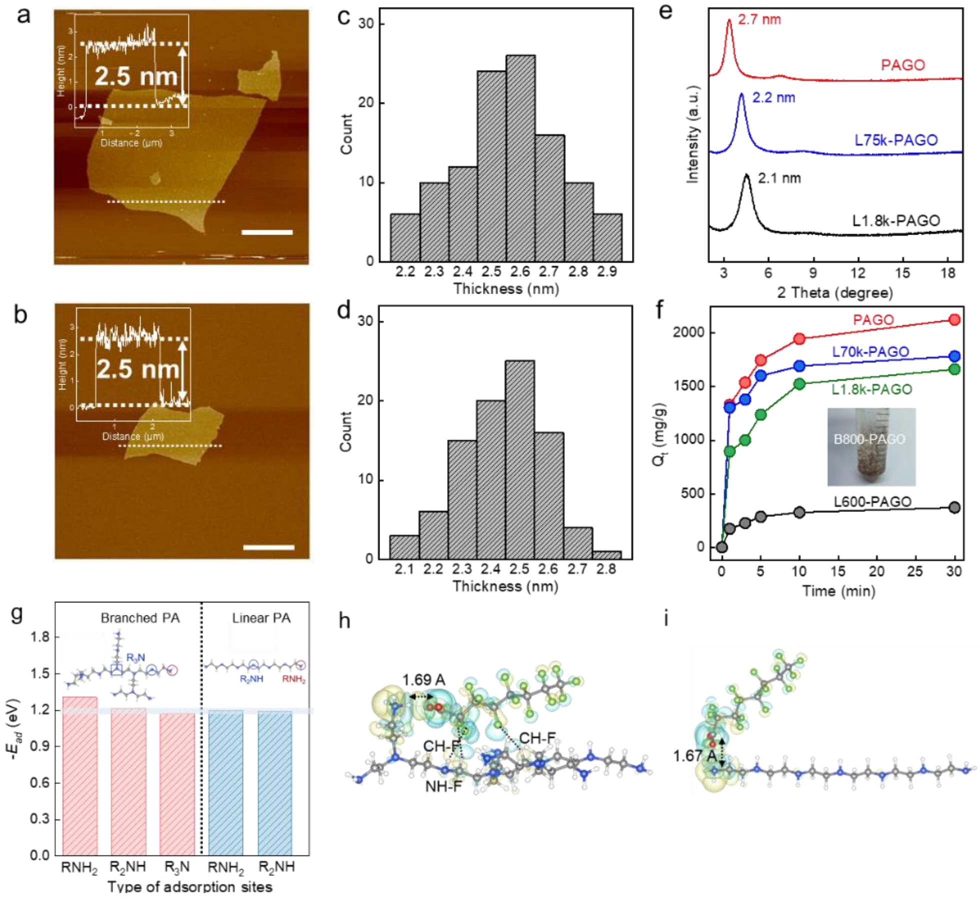

Fig. S4 Influence of molecular weight and molecular structure on the prepared PAGO and its adsorption performance. AFM images of (a) L70k-PAGO and (b) L1.8k -PAGO, insets in the upper panels are the height profile of the corresponding nanosheet measured along the white dashed line. Scale bars of a and b are 1 μm. (c) and (d) the thickness statistics measured from more than fifty nanosheets of (c) L70k-PAGO and (d) L1.8k -PAGO. (e) XRD (X-ray diffraction) patterns of PAGO, L70k-PAGO and L1.8k-PAGO. (f) The PFOA adsorption kinetics by PAGO, L70k-PAGO, L1.8k-PAGO and L600-PAGO. Inset is the picture of agglomerated B800-PAGO. (g) Calculated -E_{ad} of PFOA on different amine groups of the branched and linear PEI. (h-i) Analyses of charge density differences between (h) PFOA and branched PA and (i) PFOA and linear PA.

Bring together, we conclude that the lower Q_e for L-PAGO is accounted for the lower coverage on the PAGO surface, and fewer interaction sites that can adsorb

PFOA molecules.

To highlight the superior adsorption capacity of PAGO, we compared PAGO with other adsorbents reported in the literature, and the data are summarized in Supplementary Table 2, which clearly shows a trade-off between the Q_e and the initial concentration of PFAS. For example, the previously reported adsorbents have Q_e for PFOS from 45 to 3067 mg/g, which decrease dramatically at low PFOS concentrations. The same trends are seen for adsorbents for the removal of PFOA and PFHxA. In comparison, PAGO had significantly higher Q_e of 3089.2 mg/g, 2181.8 mg/g, and 1453.5 mg/g for the adsorption of PFOS, PFOA, and PFHxA, respectively, at a concentration of 10 mg/L, indicating its superior adsorption performance.

Table S2 Comparison of the PFAS adsorption capacity (Q_e) of previously reported adsorbents and PAGO

Adsorbent	Types of PFAS	Initial concentration of PFAS (mg/L)	Q_e (mg/g)	Equilibrium time (h)	Reference
PAF-1-NDMB	PFOS	600	2381	8	[6]
IRA67		500	3067	48	[7]
IRA958		500	2615	48	[7]
GAC		250	255	168	[8]
AI400		250	180	168	[8]
PAC		250	345	4	[8]
MOF		1	45	0.17	[9]
PAGO		10	3089.2	0.17	This work
PAF-1-NDMB	PFOA	600	2000	8	[6]
GAC		250	157	168	[8]
AI400		250	1403	168	[8]
PAC		200	327	4	[8]
Hydrotalcite		1000	2567	48	[10]
MIL-101(Cr)-QDMEN		1000	753	1	[11]
P(TMA_x-*co*-$TMPMA_{1-x}$)-CNT		414	1199.3	0.5	[12]
MWCNTs@MIPs		20	12.4	1.3	[13]
β-CD Polymer Network		12	34	24	[14]
Amberlite XAD 4		5	88	10	[15]
GO-PEI		50	302	5	[16]
PAGO		1	358.6	0.08	This work
		10	2181.8	0.17	This work

CWAC	PFHxA	250	553.08	24	[17]
IRA910		157	1099	24	[18]
IRA67		40	36	12.5	[19]
PAGO		10	1453.5	0.05	This work

2.3 PFOA removal efficiency by PAGO

To determine the size of PFOA, we used the visualization for electronic and structural analysis (VESTA) software to measure the length of PFOA, as shown in Fig. S5. The length of a PFOA molecule is estimated to be ~1.1 nm.

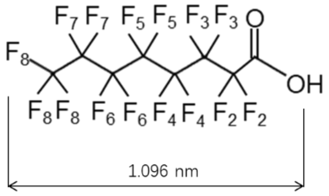

Fig. S5 The size of a PFOA molecule estimated by VESTA.

To measure the PFOA removal efficiency, a 0.2 mL PAGO dispersion (0.5 mg/mL) was added to 10 mL of PFOA solution (10 mg/L). The mixture was stirred, and sampled at different times (0, 1 minute, 3 minutes, 5 minutes and 10 minutes). The sampled mixture was filtered through a membrane filter (pore size ~0.22 μm), and the PFOA concentrations in the filtrates were analyzed by HPLC, calculated using equation (6) in Supplementary Section 2. Consistent with the observed rapid adsorption kinetics, the removal efficiency was 95.3% and ~98.8% for 1 and 10 minutes, respectively (Fig. S6a).

To examine the PFOA removal efficiency with an interfering substance, we chose humic acid (HA) which is frequently found in water and used for typical PFAS adsorption studies[20]. In this case, a mixed aqueous solution of HA and PFOA was prepared (30 ml, the concentration of HA and PFOA in the mixed solution were 5 mg/L and 10 mg/L). 1.2 mL of the 0.5 mg/mL PAGO dispersion was added to the mixed solution, and its removal efficiency was determined using the procedure mentioned above. We find a similar rapid and ~100% removal efficiency even with co-existing HA, suggesting the strong adsorption of PAGO (Fig. S6b).

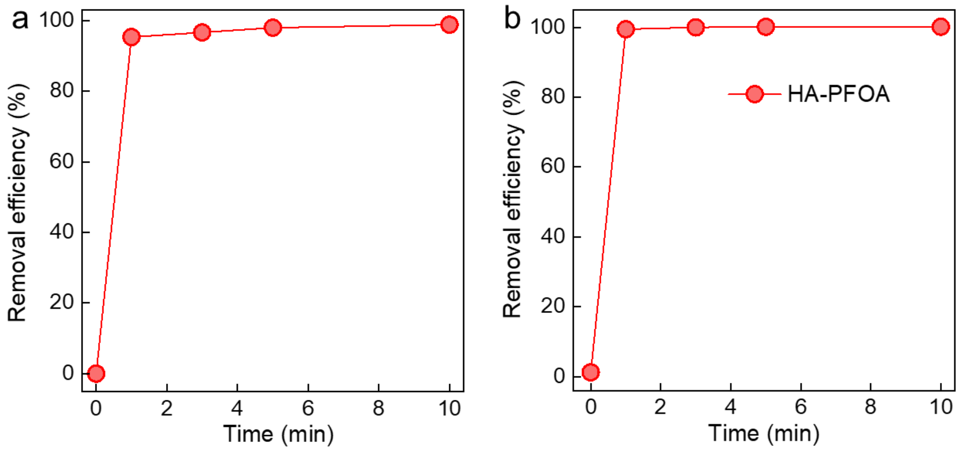

Fig. S6. (a) Removal efficiency of PFOA at 10 mg/L. (b) PFOA removal efficiency with the interfering HA solution.

2.4 Adsorption mechanism

To verify whether the adsorption kinetics could be described by an intraparticle diffusion model, we plotted the $Q_t \sim t^{1/2}$ to fit with the model expressed as Eq. (7)

$$Q_t = K_p t^{1/2} + b \qquad (7)$$

where t is adsorption time (min) and Q_t is the amount of PFOA adsorbed by PAGO (mg/g) at time t. K_p is the rate constant, and b is a constant. As shown in Fig. S7a, Q_t versus $t^{1/2}$ significantly deviates from a linear relationship, indicating that intraparticle diffusion is absent for the studied 2D PAGO adsorbent.

For clarifying the Q_e^m data, we added Table S3 to provide the calculated value for Q_e^m.

Table S3 Q_e^m of PFAS with different M_w

PFASs	Q_e (mg/g)	M_w (g/mol)	Q_e^m (mmol/g)
**PFOS**	3089.198	500.13	6.18
**PFOA**	2181.845	414.07	5.27
**PFH$_x$A (C$_6$)**	1453.505	314	4.63
**PFBA (C$_4$)**	602.051	214.04	2.81
**PFP$_r$A (C$_3$)**	225.003	164.03	1.37

Furthermore, we have fitted the adsorption kinetics with first-order and second-order adsorption respectively. As shown in Fig. S7b, the adsorption kinetics of PFAS in this study were best described by the pseudo-first-order model (R^2=0.99) rather than the pseudo-second-order model (R^2=0.96).

As noted in studies of adsorption mechanisms on homogeneous surfaces, pseudo-first-order models are typically applicable when adsorption occurs via physical interactions (e.g., electrostatic forces) without significant chemical bonding

or pore diffusion limitations[21], while, pseudo-second-order kinetics often describe systems where chemisorption (e.g., covalent bonding) or site saturation effects dominate[22].

A good fit with pseudo-first-order models is consistent with the mechanism study. Briefly, the interactions between PAGO and PFAS are identified as physical adsorption including electrostatic interaction, hydrogen bond and hydrophobic-hydrophobic interaction, and a 2D structure of PAGO is not porous excluding pore diffusion limit.

2.5 Spectroscopic analysis of PAGO after PFOA adsorption.

ATR-FTIR was used to analyze the chemical shift of PAGO after its adsorption of PFOA (Fig. S7c). It is found that the adsorption peak assigned to the amine group (N-H) has blueshifted by ~23.9 wavenumbers, which is generally believed to be associated with the formation of hydrogen bonds, indicating the formation of hydrogen bonds between the PAGO and PFOA[23].

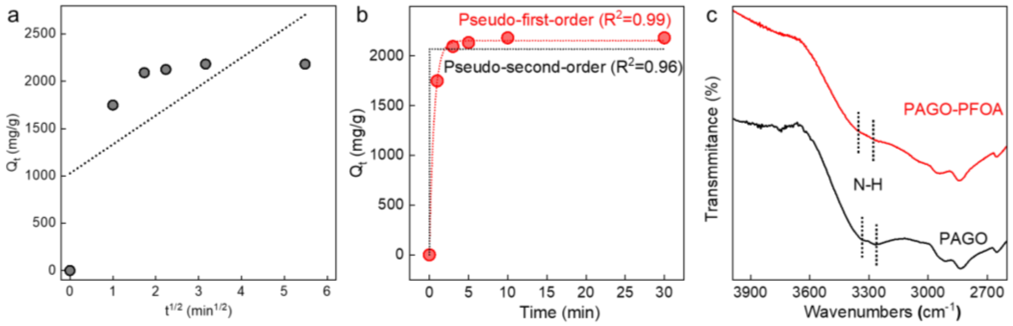

Fig. S7. (a) PFOA (10 mg/L) adsorption kinetics fitted with an interparticle diffusion model. (b) Adsorption kinetics of PFAS were described by the pseudo-first-order model and the pseudo-second-order model. (c) ATR-FTIR spectra of PAGO before (black line) and after PFOA adsorption (red line).

2.6 Measurement methods for PFAS concentrations

The concentration of PFOA (1-100 mg/L) was measured by HPLC (Shimadzu-20 A) equipped with a UV detector (the detection wavelength was 210 nm) and an inert-sustain AQ-C18 column (4.6 I.D. × 150 mm, 5 μm). The column temperature was 40 ℃. Acetonitrile and 0.1 wt % H_3PO_4 (1:1) were used as the mobile phases and the flow rate was set at 0.6 mL/min in PFOA detection.

Concentrations of PFAS ranging from 1 to 1000 μg/L were measured on LC-MS/MS spectrometry (MS-API3200, Shimadzu), and equipped with an Agilent Eclipse Plus C18 column (2.1 mm × 100 mm). The mobile phase was 2 mM

ammonium acetate solution (phase A) and methanol (phase B) at a flow rate of 0.3 mL/min and the injection volume was 10 μL.

Ultra-low concentrations of PFOA (10 ng/L-10 μg/L) were measured by UHPLC (Agilent 1290)-MS (Agilent 6470) with an Agilent Eclipse Plus C-18 narrow bore (2.1 mm×100 mm, 1.8 μm) column with a maintained temperature of 40 ℃. The flow rate was 0.3 mL/min. Chromatographic separation was achieved on solvent A (5 mM ammonium acetate in water) and solvent B (acetonitrile). The separation gradient method used was 0-5 min (holding at 5% B), 5–6 min (100%), 6 min (100%), and post-run for 3 min. The drying gas temperature and flow for MS were 300 ℃ and 7 L/min, respectively. The sheath gas temperature and flow for MS were 350 ℃ and 11 L/min, respectively. The Nebulizer pressure was 35 psi and the capillary voltage was 2500 V.

2.7 Theoretical calculation of PFOA adsorption by PAGO

Charge density difference after PFOA adsorption and an IGMH (independent gradient model based on Hirshfeld partition method) iso-surface were calculated by VASP-5.4.4 and CP2K-2022.1 software.

IGMH is a newly developed tool based on the independent gradient model (IGM) for visualizing intermolecular interactions[24,25]. In IGMH analysis, the inter-fragment interactions can be revealed by a δg^{inter} function according to equation (3) either by drawing δg^{inter} iso-surfaces or δg^{inter} vs. $sign(\lambda_2)\rho$ scattering graph. $sign(\lambda_2)\rho$ is the product of ρ and the sign of λ_2 (i.e., positive λ_2 usually corresponds to a repulsive interaction, while negative λ_2 usually corresponds to an attractive interaction), where λ_2 is the second largest eigenvalue of Hessian matrix of ρ. The value $sign(\lambda_2)\rho$ can help identify the type and reflect the strength of interactions in IGMH analysis[26,27]. Specifically, small $sign(\lambda_2)\rho$ around zero can be attributed to weak interactions such as the van der Waals interactions. Negative $sign(\lambda_2)\rho$ with relatively larger ρ values (around 0.02 to 0.05 a.u.) are usually corresponded to prominent attractive interactions such as hydrogen/halogen bond interactions of medium strength or ionic bond interactions. When ρ is larger than 0.05 a.u., the negative $sign(\lambda_2)\rho$ could be attributed to the covalent bond interaction.

$$\delta g^{\text{inter}}(\mathbf{r}) = g^{\text{IGMH, inter}}(\mathbf{r}) - g^{\text{inter}}(\mathbf{r}) \quad (3)$$

in which $g^{\text{IGMH,inter}}$ is the sum of the magnitude of density gradient of all fragments, while g^{inter} is the magnitude of the superposition of density gradient of all fragments, that is

$$g^{\text{IGMH, inter}}(\mathbf{r}) = \sum_{A} \left| \sum_{i \in A} \nabla \rho_i^{\text{Hirsh}}(\mathbf{r}) \right| \quad (4)$$

$$g^{\text{inter}}(\mathbf{r}) = \left| \sum_{A} \sum_{i \in A} \nabla \rho_i^{\text{Hirsh}}(\mathbf{r}) \right| \quad (5)$$

where *A* loops over all fragments, and *i* loops all atoms in the corresponding fragment. ρ_i^{Hirsh} is the electron density of atom *i* according to the Hirshfeld partition method[28].

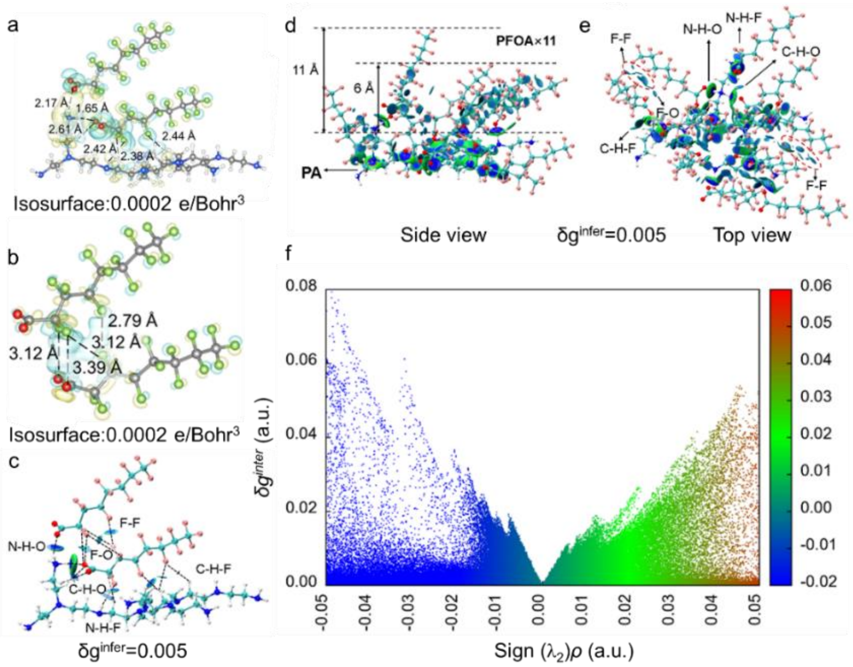

Fig. S8 Theoretical understanding of PFOA adsorption by PAGO. Analysis of charge density differences between (a) PFOA and PA and (b) PFOA and PFOA. IGMH analysis of (c) two and (d-e) eleven PFOA molecules adsorbed on PA, (d) and (e) are side and top views respectively. (f) IGMH scatter graph for interaction analysis based on eleven PFOA molecules adsorbed on PA.

Supplementary Section 3. Clean up of PFOA at environmentally-relevant and practically usable settings

In the first experiment, we tested PAGO for its removal of PFOA from Xiaoqing River water. Consistent with the lab adsorption test, even at an environment-relevant concentration as low as ~160 μg/L, PAGO remains highly efficient and removes 99.3% of PFOA in 1 minute (Fig. S9).

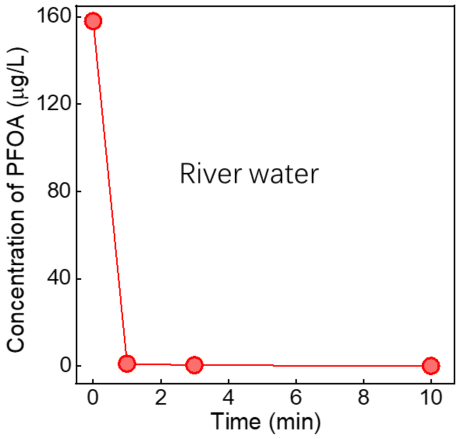

Fig. S9. Adsorption performance of PAGO in removing practical contaminated water (initial concentration with 158 ng/L).

In the second experiment, considering that a practically usable adsorbent should allow a continuous adsorption process. We have tried a fixed-bed experiment for the continuous removal of PFOA (Fig. S10a). The 70 ml chromatographic columns (DB04, inner diameter = 26.8 mm) were filled with ~9 g of pristine degreased cotton, and PAGO-coated cotton (Fig. S10b) respectively. In detail, cotton was soaked in PAGO dispersion (0.5 mg/mL) overnight and vacuum dried at 45 ° for 3 days. The mass of PAGO on the surface of cotton is ~103 mg (determined by comparing the weight difference before and after PAGO modification). The PFOA solution (500 μg/L) was pumped into the column (9.8 mL/min) and the filtrates were collected to determine the PFOA concentration using LC-MS/MS spectrometry. As shown in Fig. S10c, coating PAGO on cotton shows significant improvements in continuous PFOA removal. Fig. S10c inset shows the corresponding removal efficiency. PAGO maintains >95% efficiency even after processing 108.9 L of PFOA-contaminated water, demonstrating its exceptional capacity for continuous operation.

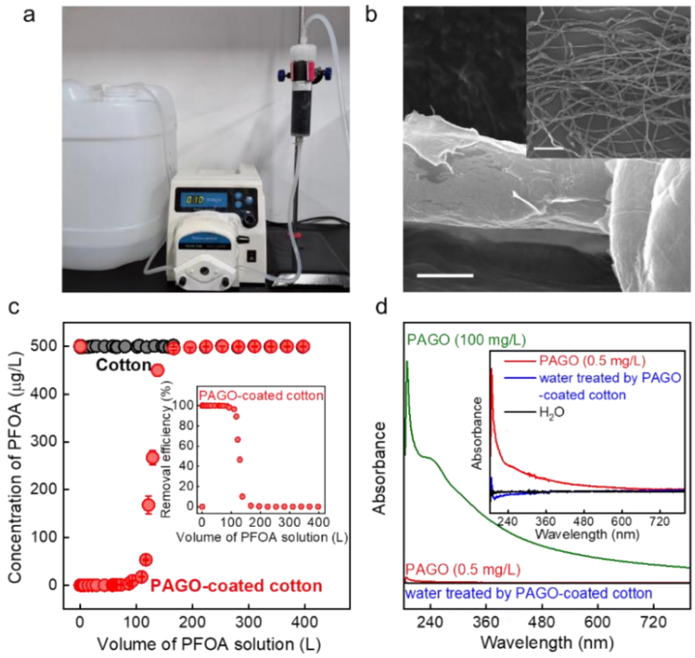

Fig. S10 The fixed-bed experiments for continuous PFOA removal. (a) The experimental setup for fixed-bed column continuous adsorption. (b) SEM image of PAGO nanosheets loaded on a cotton fiber. Scale bar of b,1 μm. Inset is the low magnification image of PAGO-coated cotton fibers, scale bar of inset, 200 μm. (c) The change of PFOA concentration in the permeate during continuous treatment of a certain volume of PFOA solution. Inset is the change of removal efficiency of PFOA with the treatment of volume of PFOA solution by PAGO-coated cotton in a fixed-bed column. (d) UV-vis spectra of PAGO dispersion with concentrations of 100 mg/L (green) and 0.5 mg/L (red), water after fixed bed treatment by PAGO-coated cotton (blue).

Considering the coating of PAGO on cotton may lead to detachment of PAGO from the cotton surface and release into the treated/purified water, we analyzed the residual PAGO in the purified water after fixed-bed treatment using Ultraviolet-visible (UV-Vis) spectroscopy (Fig. S10d). We found UV-Vis spectroscopy is capable of detecting a PAGO concentration of as low as 0.5 mg/L in water, and the analysis of water treated by PAGO-coated cotton shows no characteristic peak of PAGO, suggesting no release of PAGO in treated water. These experimental results show that PAGO-coated cotton fiber is safe without release to water, therefore is promising for use in a fixed-bed, continuous and separation-free water treatment setting with a high PFAS removal efficiency.

Supplementary Section 4. Desorption-enrichment-photocatalytic degradation of PFOA

For the photocatalytic degradation, 10 mg In_2O_3 was added as the photocatalyst in a 200 mL PFOA aqueous solution with initial concentrations (C_0) of 0.5, 1, 5, and

10 mg/L. Before UV irradiation, the mixture was stirred in the dark for 30 minutes to reach adsorption equilibrium. UV irradiation was applied using a 10 W, 254 nm UV lamp with an average irradiation intensity of ~35 W/m^2. After 20 minutes of irradiation, 2 mL suspensions were sampled and filtered through a membrane with an average pore size of 0.22 μm, and the PFOA concentration in the filtrate (C) was determined using LC-MS/MS system.

As shown in Fig. S11, we find a high degradation efficiency when the initial PFOA concentration is high (5 and 10 mg/L), reaching 93%. Such efficiency becomes low (<50%) at low concentrations (1 and 0.5 mg/L). This is unsurprising, and possibly because of an increased diffusion barrier for PFOA to reach the catalyst surface. Based on this result, we proposed to use desorption and concentration enrichment for a high photodegradation efficiency as shown in Fig. 4d the main text.

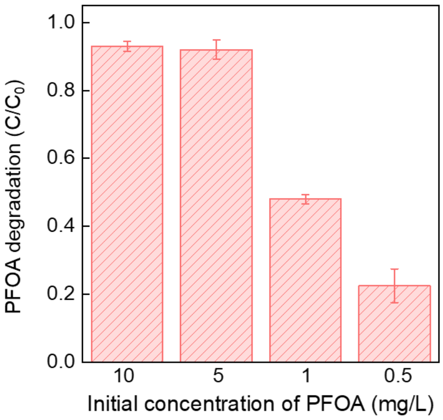

Fig. S11 The photocatalytic degradation performance of PFOA with different concentrations after 20-minute UV irradiation.

Supplementary Section 5. Material characterization and calculation

5.1 Material characterization

Zeta potential measurements were performed using Zetasizer Lab (Malvern Instruments, UK). Infrared spectra were recorded on a Bruker TENSOR 37 FTIR spectrometer equipped with an ATR accessory. Solid-state 19F NMR was conducted on an Agilent 600 M system with a 4.0 mm MAS probe. UV-Vis absorption spectra were recorded on a UV-Vis spectrophotometer (JASCO, V-760). The thickness of GO, PAGO and adsorbed PFOA was measured by AFM using the tapping mode (Bruker Dimension Icon, tapping mode). XRD was performed using a Bruker D8 Advance X-ray transmission diffractometer with Cu Kα radiation. The STEM image of PAGO was obtained by a cold-field-emission spherical aberration-corrected transmission

electron microscope (Thermo Fisher Scientific, Spectra 300) operated at 200 kV.

5.2 Adsorption energy calculation

All adsorption energies were calculated by the Vienna Ab Initio Simulation Package (VASP-5.4.4) using the Perdew-Burke-Ernzerh (PBE) exchange-correlation functional. The core electrons for the calculation models were treated with the projector–augmented wave (PAW) method with an energy cutoff of 600 eV and a Gaussian smearing width of 0.05 eV. The implicit solvent model was considered in all DFT calculations. Before energy calculation, the adsorption models were optimized with convergence thresholds of 10^{-5} eV for electronic energy and 0.05 eV/Å for force. The GO structure for the PAGO model was a $30 \times 26 \times 40$ Å^3 orthorhombic cell and was adapted from a previous report[29].

5.3 Intermolecular interaction analysis

The intermolecular interactions contributing to PFOA adsorption on PAGO were analyzed by charge density differences using VASP calculations and IGMH function[30]. For the VASP calculation, the wave function was calculated by CP2K-2022.1 software[31] with Multiwfn program[32] and visualized by VMD 1.9.3 software[33]. For IGMH analysis, all DFT calculations were conducted on the PBE-D3(BJ)/6-311G** level[34-36] with plane waves expanded to a 400 Ry (the unit of cutoff energy) absolute energy cutoff and a 55 Ry relative cutoff using a $30 \times 26 \times 40$ Å^3 orthorhombic cell.

References

1. Wan S, Peng J, Li Y, Hu H, Jiang L, Cheng Q. Use of synergistic interactions to fabricate strong, tough, and conductive artificial nacre based on graphene oxide and chitosan. *ACS Nano* **9**, 9830-9836 (2015).
2. Andreeva DV, *et al.* Two-dimensional adaptive membranes with programmable water and ionic channels. *Nat. Nanotechnol.* 16, 174-180 (2021).
3. Lützenkirchen J, Preočanin T, Kovačević D, Tomišić V, Lövgren L, Kallay N. Potentiometric titrations as a tool for surface charge determination. *Croat. Chem. Acta* **85**, 391-417 (2012).
4. Craciun BF, Gavril G, Peptanariu D, Ursu LE, Clima L, Pinteala M. Synergistic Effect of Low Molecular Weight Polyethylenimine and Polyethylene Glycol Components in Dynamic Nonviral Vector Structure, Toxicity, and Transfection Efficiency. *Molecules* **24**, 1460 (2019).
5. Henderson Jr WA, Schultz CJ. The nucleophilicity of amines. *J. Org. Chem.* **27**, 4643-4646 (1962).
6. Liu X, *et al.* Installation of synergistic binding sites onto porous organic polymers for efficient removal of perfluorooctanoic acid. *Nat. Commun.* **13**, 2132 (2022).
7. Deng S, Yu Q, Huang J, Yu G. Removal of perfluorooctane sulfonate from wastewater by anion exchange resins: Effects of resin properties and solution chemistry. *Water Res.* **44**, 5188-5195 (2010).
8. Yu Q, Zhang R, Deng S, Huang J, Yu G. Sorption of perfluorooctane sulfonate and perfluorooctanoate on activated carbons and resin: Kinetic and isotherm study. *Water Res.* **43**, 1150-1158 (2009).
9. Li Y, *et al.* A mesoporous cationic thorium-organic framework that rapidly traps anionic persistent organic pollutants. *Nat. Commun.* **8**, 1354 (2017).
10. Rattanaoudom R, Visvanathan C, Boontanon SK. Removal of concentrated PFOS and PFOA in synthetic industrial wastewater by powder-activated carbon and hydrotalcite. *J. Water Sustain* **2**, 245-258 (2012).
11. Liu K, Zhang S, Hu X, Zhang K, Roy A, Yu G. Understanding the adsorption of PFOA on MIL-101(Cr)-based anionic-exchange metal-organic frameworks: comparing DFT calculations with aqueous sorption experiments. *Environ. Sci. Technol.* **49**, 8657-8665 (2015).
12. Kim K, *et al.* Molecular tuning of redox‐copolymers for selective electrochemical remediation. *Adv. Funct. Mater.* **30**, 2004635 (2020).
13. Cao F, Wang L, Yao Y, Wu F, Sun H, Lu S. Synthesis and application of a highly selective molecularly imprinted adsorbent based on multi-walled carbon nanotubes for selective removal of perfluorooctanoic acid. *Environ. Sci. Water Res. Technol.* **4**, 689-700 (2018).
14. Xiao L, Ling Y, Alsbaiee A, Li C, Helbling DE, Dichtel WR. β-Cyclodextrin polymer network sequesters perfluorooctanoic acid at environmentally relevant concentrations. *J. Am. Chem. Soc.* **139**, 7689-7692 (2017).
15. Senevirathna S, *et al.* Adsorption of four perfluorinated acids on non ion

exchange polymer sorbents. *Water Sci. Technol.* **63**, 2106-2113 (2011).

16. Lei, X. *et al.* Removal of perfluorooctanoic acid via polyethyleneimine modified graphene oxide: Effects of water matrices and understanding mechanisms. *Chemosphere* **308**, 136379 (2022).
17. Shi Y, *et al.* Confined water–encapsulated activated carbon for capturing short-chain perfluoroalkyl and polyfluoroalkyl substances from drinking water. *Proc. Natl. Acad. Sci. U.S.A.* **120**, e2219179120 (2023).
18. Maimaiti, A. *et al.* Competitive adsorption of perfluoroalkyl substances on anion exchange resins in simulated AFFF-impacted groundwater. *Chem. Eng. J.* **348**, 494-502 (2018).
19. Du, Z. *et al.* Removal of perfluorinated carboxylates from washing wastewater of perfluorooctanesulfonyl fluoride using activated carbons and resins. *J. Hazard. Mater.* **286**, 136-143 (2015).
20. Deng S, Bai RB. Aminated polyacrylonitrile fibers for humic acid adsorption: behaviors and mechanisms. *Environ. Sci. Technol.* **37**, 5799-5805 (2003).
21. Musah M, Azeh Y, Mathew J T, et al. Adsorption kinetics and isotherm models: a review. CaJoST, 4(1), 20-6 (2022).
22. Zeng Z, Li Q, Yan J, et al. The model and mechanism of adsorptive technologies for wastewater containing fluoride: a review. Chemosphere, 340, 139808 (2023).
23. Fang Q, Chen B, Zhuang S. Triplex blue-shifting hydrogen bonds of ClO4–··H–C in the nanointerlayer of montmorillonite complexed with cetyltrimethylammonium cation from hydrophilic to hydrophobic properties. *Environ. Sci. Technol.* **47**, 11013-11022 (2013).
24. Lu T, Chen Q. Independent gradient model based on Hirshfeld partition: A new method for visual study of interactions in chemical systems. *J. Comput. Chem.* **43**, 539-555 (2022).
25. Lefebvre C, Rubez G, Khartabil H, Boisson J-C, Contreras-García J, Hénon E. Accurately extracting the signature of intermolecular interactions present in the NCI plot of the reduced density gradient versus electron density. *Phys. Chem. Chem. Phys.* **19**, 17928-17936 (2017).
26. Johnson ER, Keinan S, Mori-Sánchez P, Contreras-García J, Cohen AJ, Yang W. Revealing noncovalent interactions. *J. Am. Chem. Soc.* **132**, 6498-6506 (2010).
27. Lu T, Chen Q. Visualization Analysis of weak interactions in chemical systems. *Comput. Theor. Chem.* **2**, 240-264 (2024).
28. Hirshfeld FL. Bonded-atom fragments for describing molecular charge densities. Theor. Chim. Acta 44, 129-138 (1977).
29. Mouhat F, Coudert F-X, Bocquet M-L. Structure and chemistry of graphene oxide in liquid water from first principles. Nat. Commun. 11, 1566 (2020).
30. Lu T, Chen Q. Independent gradient model based on Hirshfeld partition: A new method for visual study of interactions in chemical systems. J. Comput. Chem. 43, 539-555 (2022).
31. Kühne TD, et al. CP2K: An electronic structure and molecular dynamics

software package-Quickstep: Efficient and accurate electronic structure calculations. J. Chem. Phys. 152, 194103 (2020).

32. Lu T, Chen F. Multiwfn: A multifunctional wavefunction analyzer. J. Comput. Chem. 33, 580-592 (2012).
33. Humphrey W, Dalke A, Schulten K. VMD: visual molecular dynamics. J. Mol. Graph. 14, 33-38 (1996).
34. Grimme S, Antony J, Ehrlich S, Krieg H. A consistent and accurate ab initio parametrization of density functional dispersion correction (DFT-D) for the 94 elements H-Pu. J. Chem. Phys. 132, 154104 (2010).
35. Grimme S, Ehrlich S, Goerigk L. Effect of the damping function in dispersion corrected density functional theory. J. Comput. Chem. 32, 1456-1465 (2011).
36. Perdew JP, Burke K, Ernzerhof M. Generalized gradient approximation made simple. Phys. Rev. Lett. 77, 3865 (1996).
